# Physiological and Transcriptomic Evaluation of Drought Effect on Own-Rooted and Grafted Grapevine Rootstock (1103P and 101-14MGt)

**DOI:** 10.3390/plants12051080

**Published:** 2023-02-28

**Authors:** Davide Bianchi, Valentina Ricciardi, Carola Pozzoli, Daniele Grossi, Leila Caramanico, Massimo Pindo, Erika Stefani, Alessandro Cestaro, Lucio Brancadoro, Gabriella De Lorenzis

**Affiliations:** 1 Dipartimento di Scienze Agrarie e Ambientali-Produzione Territorio e Agroenergia, Università degli Studi di Milano, Via G. Celoria 2, 20133 Milano, Italy; 2 Dipartimento di Scienze Farmacologiche e Biomolecolari, Università degli Studi di Milano, Via Balzaretti 9, 20133 Milano, Italy; 3 Fondazione E. Mach, Centro Ricerca e Innovazione, Via E. Mach 1, 38010 San Michele all’Adige, TN, Italy

**Keywords:** ABA, avoidance, Cabernet Sauvignon, gas exchange, tolerance, water stress-response

## Abstract

Grapevines worldwide are grafted onto *Vitis* spp. rootstocks in order to improve their tolerance to biotic and abiotic stresses. Thus, the response of vines to drought is the result of the interaction between the scion variety and the rootstock genotype. In this work, the responses of genotypes to drought were evaluated on 1103P and 101-14MGt plants, own-rooted and grafted with Cabernet Sauvignon, in three different water deficit conditions (80, 50, and 20% soil water content, SWC). Gas exchange parameters, stem water potential, root and leaf ABA content, and root and leaf transcriptomic response were investigated. Under well-watered conditions, gas exchange and stem water potential were mainly affected by the grafting condition, whereas under sever water deficit they were affected by the rootstock genotype. Under severe stress conditions (20% SWC), 1103P showed an “avoidance” behavior. It reduced stomatal conductance, inhibited photosynthesis, increased ABA content in the roots, and closed the stomata. The 101-14MGt maintained a high photosynthetic rate, limiting the reduction of soil water potential. This behavior results in a “tolerance” strategy. An analysis of the transcriptome showed that most of the differentially expressed genes were detected at 20% SWC, and more significantly in roots than in leaves. A core set of genes has been highlighted on the roots as being related to the root response to drought that are not affected by genotype nor grafting. Genes specifically regulated by grafting and genes specifically regulated by genotype under drought conditions have been identified as well. The 1103P, more than the 101-14MGt, regulated a high number of genes in both own-rooted and grafted conditions. This different regulation revealed that 1103P rootstock readily perceived the water scarcity and rapidly faced the stress, in agreement with its avoidance strategy.

## 1. Introduction

Drought is a relevant environmental factor affecting grape quality and productivity. The response of a grapevine to drought involves several physiological processes in order to preserve the hydraulic conductance in a soil–plant–atmosphere continuum. Adaptation to drought depends on the intensity and the extension of the water deficit [1,2,3]. Severe water deficit leads to plant water potential drop, reducing hydraulic conductance, which in turn can result in xylem embolisms [2,4,5]. These conditions affect vine vegetative growth and grape ripening [6].

The drop in water potential can be buffered by the early reduction of stomatal conductance in response to water deficit [7,8]. Stomatal closure under drought conditions is induced by hydraulic and hormonal signalling [9]. The main hormone involved in the stomatal closure is abscisic acid (ABA). ABA is produced by both roots and leaves under soil water deficit in response to the reduction of water potential, which reaches guard cells through the xylem sap. The ABA accumulation in guard cells leads to an increase of reactive oxygen species (ROS), which causes a loss of turgor through the regulation of potassium/calcium balance [10,11]. Due to the peculiar structure of guard cells, the turgor loss leads to the closure of stomata. Although stomatal closure preserves a favourable water balance, the carbon assimilation through the photosynthetic activity can be considerably limited [12].

On top of that, photosynthesis in plants can be affected by non-stomatal limitations. Under severe and prolonged water stress, stomatal control of the photosynthetic rate becomes progressively less effective and mesophyll metabolism is affected. This is shown as a reduction of the photosynthetic capacity due to the decreased synthesis of ribulose biphosphate, the reduction of Rubisco activity, and carboxylation efficiency [13].

As a consequence of altered photosynthetic activity, starch reserves are consumed to overcome the reduced carbon assimilation, in order to provide sugars for primary metabolism and derived compounds to mitigate water deficit [14]. The metabolites produced in response to a water deficit are generally hydrophilic compounds, which affect osmotic potential to restore the water uptake, such as sucrose, fructose, mannitol, glycerol, and proline [15]. Other compounds include carotenoids and flavonoids, which are involved in the detoxification of ROS excess caused by the stress conditions [16].

Grapevines (*Vitis vinifera*) worldwide are grafted onto rootstocks since the end of the 19th century, to counteract the damage of phylloxera (*Daktulospharia vitifoliae*). This aphid attacks the root system of *V. vinifera* causing the death of the plant, however, most of the American *Vitis* species are not susceptible [17]. Thus, American *Vitis* species and their hybrids have been used as rootstocks to confer resistance to the *V. vinifera* varieties. The interaction between scion and rootstock also affects tolerance to abiotic stresses such as mineral nutrition, limestone, salinity, and drought [15,18,19].

Grapevines are mainly grown in temperate areas and traditionally non-irrigated, due to an adaptation to limited water availability conditions. Nevertheless, a large variability in drought tolerance among *Vitis* genotypes has been detected [6]. The interest in understanding the physiological mechanisms involved in drought tolerance is further enhanced by new conditions imposed by climate change [6,20]. Based on the increase of arid and semi-arid conditions in several viticultural areas, the selection of new rootstocks with high water use efficiency, plant growth capacity, and scion adaptability represents an important strategy to face the negative effects of drought [21,22,23]. The rootstock genotype affects the response of the scion to drought in several ways: controlling the gas exchange and the water use efficiency at the leaf level [9,21,24,25]; determining the size and the depth of the root system, impacting water uptake during water deficit [26]; and sensing and responding to water deficit signals [27,28,29]. For example, rootstock 1103P induces stomatal closure to reduce water loss during dry periods and increases water uptake, thus growing a wider and deeper root system than rootstock 101-14MGt [26].

At the transcriptomic level, drought induces the modulation of genes involved in different pathways: (i) phenylpropanoid pathway, such as resveratrol and flavonoid biosynthetic genes; (ii) ABA metabolism; (iii) carbohydrate metabolism; (iv) stress-responsive pathway, such as the nucleotide-binding domain/leucine-rich repeat (NBS-LRR) class and several pathogenesis-related proteins (PRPs); (v) signal transduction; and (vi) photosynthesis [22,30]. A strong modulation of transcription factors, such as MYB, NAC, bHLH, and HSF, has been observed in grapevine plants subjected to drought [31].

Although the influence of rootstock genotypes on scion physiological performance has been strongly investigated, the comprehension of grafted status and scion genotype impact on rootstock performance under water stress conditions is still less debated. In this work, drought has been evaluated on own-rooted and grafted vines. Two rootstock genotypes, a drought-susceptible (101-14MGt) and a drought-tolerant (1103P), own-rooted and grafted with Cabernet Sauvignon, were subjected to a gradual water shortage in semi-controlled environmental conditions and their phenotypical and transcriptomic responses have been recorded.

## 2. Results

### 2.1. Phenotypic Response of Own-Rooted Plants to Water Deficit

**Figure 1:**
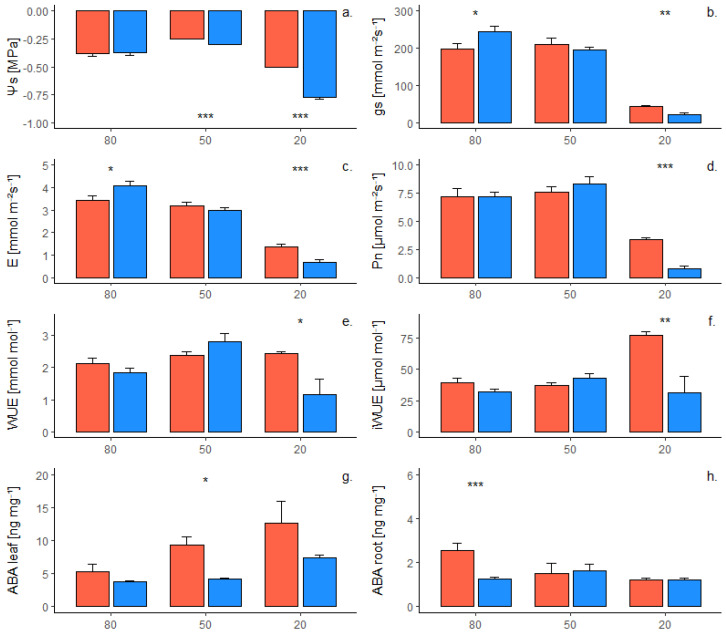
Kinetics of two own-rooted rootstock genotypes (1103P, in blue and 101-14MGt, in red) under decreasing water availability for (**a**) stem water potential, (**b**) stomatal conductance, (**c**) transpiration, (**d**) carbon assimilation, (**e**) instantaneous water use efficiency, (**f**) intrinsic water use efficiency, and ABA concentration in (**g**) leaves and (**h**) roots. Dots represent mean values of three biological repetitions and bars are the standard errors of means. Significant differences were reported for 0.01 ≤ *p* ≤ 0.05 (*), 0.001 ≤ *p* ≤ 0.01 (**) and *p* ≤ 0.001 (***).

The response of the two own-rooted rootstock genotypes in terms of water potential, gas exchange, and ABA concentration in leaves and roots was reported in Figure 1. At 80% SWC (soil water content), rootstock 1103P reported higher gs (stomatal conductance) and E (transpiration) than 101-14MGt (Figure 1b,c) and a lower concentration of ABA in the roots (Figure 1h). Slight differences occurred at 50% SWC, whereas at 20% the 101-14MGt rootstock reported higher levels of Ψs (stem water potential, Figure 1a), gs (Figure 1b), E (Figure 1c), Pn (carbon assimilation by photosynthetic activity, Figure 1d), WUE (water use efficiency, Figure 1e), and iWUE (intrinsic WUE, Figure 1f) than 1103P. At the end of the experiment, no differences were found between the two genotypes in terms of leaf biomass (data not shown).

A significant regression was found between Ψs and gs for own-rooted 1103P, reporting a coefficient of determination of 0.58. The same relation was shown by 101-14MGt, but a lower R^2^ was reported (0.23). Rootstock 1103P also reported significant regressions between Ψs and gs, Pn and WUE, whereas none of these regressions were found for 101-14MGt (Table 1). Furthermore, the concentration of ABA in leaves significantly increased at decreasing Ψs for 1103P (R^2^ = 0.41), and no significant trend was found for 101-14MGt. The concentration of ABA in roots did not depend on Ψs for any of the analyzed own-rooted genotypes (Table 1). Regression coefficients were not statistically different between the two genotypes. All regressions can be found in Supplementary material S1.

### 2.2. Phenotypic Response of Grafted Plants to Water Deficit

**Figure 2:**
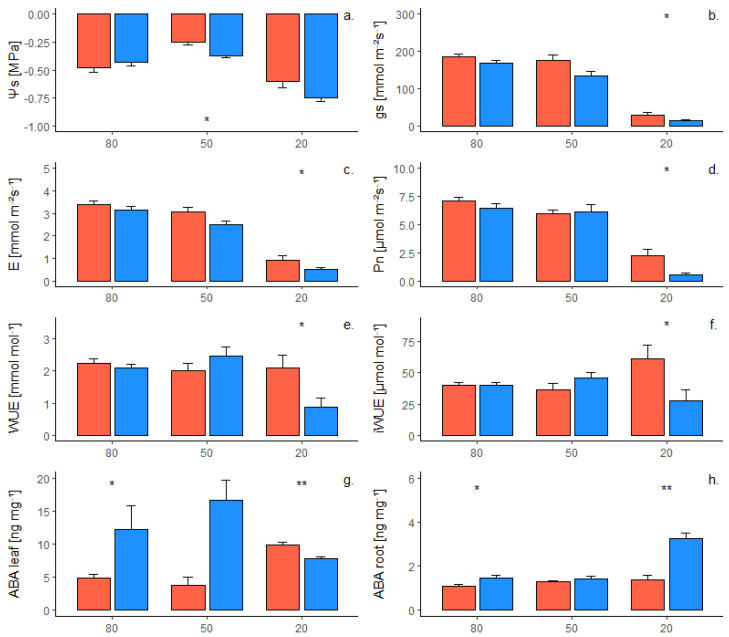
Kinetics of Cabernet Sauvignon grafted onto two rootstock genotypes (1103P, in blue and 101-14MGt, in red) under decreasing water availability for (**a**) stem water potential, (**b**) stomatal conductance, (**c**) transpiration, (**d**) carbon assimilation, (**e**) instantaneous water use efficiency, (**f**) intrinsic water use efficiency, and ABA concentration in (**g**) leaves and (**h**) roots. Dots represent mean values of three biological repetitions and bars are the standard errors of means. Significant differences were reported for 0.01 ≤ *p* ≤ 0.05 (*) and 0.001 ≤ *p* ≤ 0.01 (**).

When Cabernet Sauvignon is grafted onto the two rootstock genotypes, slight differences occurred at 80% SWC. At this SWC level, ABA concentration in both leaves and roots was significantly higher for plants grafted onto 1103P (Figure 2g,h). At 50% SWC, the grafting combination with 101-14MGt significantly increased Ψs (Figure 2a), but no other differences were observed. Finally, at 20% SWC, scion grafted onto 101-14MGt were characterized by higher levels of gs (Figure 2b), E (Figure 2c), Pn (Figure 2d), WUE (Figure 2e), iWUE (Figure 2f), and ABA_leaf_ (Figure 2g) than scion grafted onto 1103P, whereas the latter reported higher concentration of ABA in the roots (Figure 2h). At the end of the experiment, no differences were found between the two grafting combinations in terms of leaf biomass (data not shown).

Similar to own-rooted conditions, the regression analysis showed a significant relation between Ψs and gas exchange for rootstock 1103P, although the coefficients of determination were generally lower in grafted plants. Likewise, the lack of significance between Ψs and gas exchange reported for own-rooted 101-14MGt was confirmed in grafted plants (Table 2).

Nevertheless, the level of ABA in leaves of Cabernet Sauvignon showed a different trend compared to un-grafted rootstocks. In particular, a significant regression was found for plants grafted onto 101-14MGt (R^2^ = 0.308), and no relation was reported for plants grafted onto 1103P. On the contrary, the level of ABA in roots was significantly related to Ψs for grafted 1103P (R^2^ = 0.32), whereas the same regression for 101-14MGt was not significant. Significant differences between the two grafting combinations were found for regression coefficients, showing that ABA in roots of 1103P increased more than 101-14MGt when grafted onto Cabernet Sauvignon (Table 2). All regressions are presented in Supplementary material S1.

### 2.3. Interaction of Genotype and Presence of Scion on Phenotypic Traits

Analysis of variance showed the relative contribution of rootstock genotype (R), grafting status (G), and their interaction (R ∗ G) for all the phenotypic traits investigated at different levels of SWC (Table 3). At 80% SWC, the largest part of the variance was explained by G for several traits, e.g., Ψs, gs, Pn, E, and iWUE. A significant effect of G was found for Ψs, Gs, E, and the concentration of ABA in both leaves and roots, which was also reported as a significant effect of R ∗ G except for Ψs, whereas the effect of R was only significant for ABA_root_. At 50% SWC, G explained the largest part of variance for gs and Pn, but a significant effect was also found for Ψs and ABA_leaf_. The traits Ψs, E, WUE, and iWUE were mainly explained by R, although a significant effect was only reported for Ψs and E. The variance of ABA_leaf_ was mainly explained by the significant R ∗ G interaction. Considering the level of 20% SWC, R became the largest source of variability for all the phenotypic traits, except for ABA_root_, showing a highly significant effect except with regards to ABA_leaf_. A significant effect of G was reported for gs, E, and ABA_root_, whereas the R ∗ G interaction was only significant for ABA_root_.

**Figure 3:**
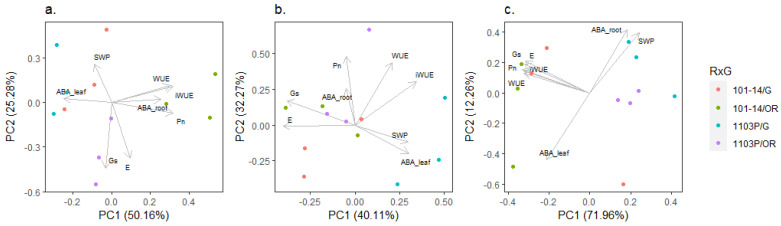
Principal component analysis of phenotypic traits under (**a**) 80% SWC, (**b**) 50% SWC, and (**c**) 20% SWC.

The relation among phenotypic traits along the different levels of SWC were highlighted by the principal component analysis, as reported in Figure 3. At 80% SWC, the two principal uncorrelated components explained the 75.44% of the total variance. The first component (PC1) explained 50.16% of the variance, and it was positively affected by Pn, WUE, iWUE, and ABA_root_, and negatively affected by ABA_leaf_. The second component (PC2) explained 25.28% of the total variance and it was related to Ψs, gs, and E. According to the first component, own-rooted rootstock genotypes are clearly separated, whereas grafted plants are grouped (Figure 3a). At 50% SWC, PC1 and PC2 explained the 40.11% and 32.27% of total variance, respectively, for a total of 72.38% of represented variance. The first component was positively affected by WUE, iWUE, Ψs, and ABA_leaf_ and negatively affected by gs and E, whereas the second component was mainly related to Pn, WUE, and iWUE. While own-rooted and grafted conditions separated for 1103P along the first component, they were not distinguished for 101-14MGt at 50% SWC (Figure 3b). At 20% SWC, two significant components were found to explain the 84.22% of total variance. The largest part of variance was explained by PC1 (71.96%), negatively affected by gas exchange (i.e., Pn, E and gs), water use efficiency (i.e., WUE and iWUE), and ABA in the leaves. PC2 was also negatively affected by ABA_leaf_ and positively affected by ABA_root_ and Ψs. According to PC1, the analyzed vines clustered for the rootstock genotype, regardless of the presence of scion (Figure 3c). According the two main components, rootstock 1103P reported a similar water status between repetitions at 50% and 20% SWC, whereas the response of 101-14MGt was more diversified between repetitions for both grafted and own-rooted vines (Figure 3b,c).

### 2.4. Transcriptomic Response of Own-Rooted and Grafted Plants to Water Deficit

The NGS technology using the HiSeq2000 platform allowed us to sequence the whole transcriptome of roots of two rootstock genotypes (1103P and 101-14MGt), as well as the leaves of two rootstock genotypes (1103P and 101-14MGt) and one cultivar (Cabernet Sauvignon plants grafted onto 1103P and 101-14MGt). An average of 57 million unique reads, ranging from 45 to 80 million, were mapped according to the grape reference transcriptome CRIBI PN40024 12X v2. The percentage of successfully mapped reads amounted to 84%, in a range from 62 to 91%. Similar patterns were obtained by the three biological repetitions, as shown by the PCA in Supplementary material S2. The two main components identified by PCA overall accounted for the 92% of the variability. Root and leaf samples were clearly split along PC1, except for two samples (i.e., 1103P:own-rooted:drought:roots:T3 and 1103P:own-rooted:well-watered:roots:T1), which were separately grouped according to PC2 and not included in following tests. The same two groups, representing the different tissue samples, were confirmed by cluster analysis and heatmaps, as shown in Supplementary material S3. Inside the root cluster, PC1 and PC2 were able to distinguish three main groups: the first one represented by the samples 1103P:own-rooted:drought:roots:T3 and 1103P:own-rooted:well-watered:roots:T1, the second one including twelve samples of mixed genotypes, and the third one separated the two genotypes, regardless of water and grafting conditions. In order to address concerns about leaf cluster, as expected, the two main groups were represented by the grafting conditions, with the two rootstocks genotypes clearly separated within the own-rooted conditions.

**Figure 4:**
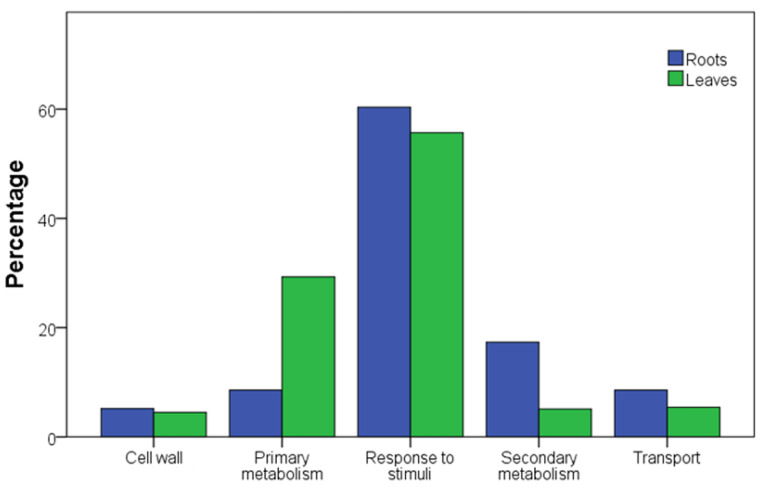
Overview of ontology categories of DEGs in 101-14MGt and 1103P roots and leaves of own-rooted and grafted plants under drought conditions. GO terms were grouped in five micro-categories: cell wall, primary metabolism, response to stimuli, secondary metabolism, and transport. All the DEGs have been used.

The differentially expressed genes (DEGs) of the two rootstock genotypes at decreasing water availability levels are summarized in Table 4 and listed as Supplementary materials S4 and S5. Under well-watered conditions (T1; SWC 80%), any significant difference was reported between genotypes or grafting conditions. The highest number of DEGs was observed at T3 (SWC 20%), especially at the root level. Regardless of the grafting condition, under severe water deficit, 1103P reported more DEGs than 101-14MGt under water deficit. The main biological processes affected by drought were investigated using a GO enrichment assay, as shown in Supplementary materials S6 and S7. Five macro-categories were identified by grouping the top 50 GO (Figure 4), with a similar distribution of DEGs between the analyzed plant tissues. The category “response to stimuli” was the most enriched GO for both roots and leaves, amounting to 60 and 55%, respectively, whereas “cell wall” was the lowest at about 5%. In leaf samples, drought affected about the 30% of genes involved in primary metabolism and 8% in secondary metabolism, whereas in root samples an opposite trend was observed, affecting 5 and 17% of genes involved in primary and secondary metabolisms, respectively.

**Figure 5:**
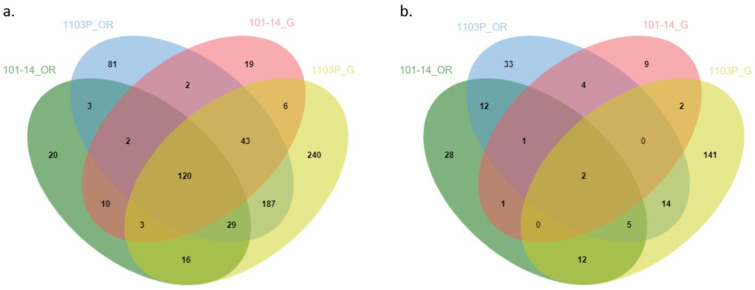
Venn diagram illustrating DEGs by roots and leaves of two grapevine rootstocks (101-14MGt and 1103P), own-rooted and grafted with Cabernet Sauvignon, at 20% soil water content (T3). Only the genes with a log2 fold change value higher than 2.0 and lower than −2.0 were viewed. (**a**) DEGs in roots of 101-14MGt and 1103P, own-rooted (OR) and grafted (G); (**b**) DEGs in leaves of 101-14MGt and 1103P, own-rooted (OR) and grafted (G).

**Figure 6:**
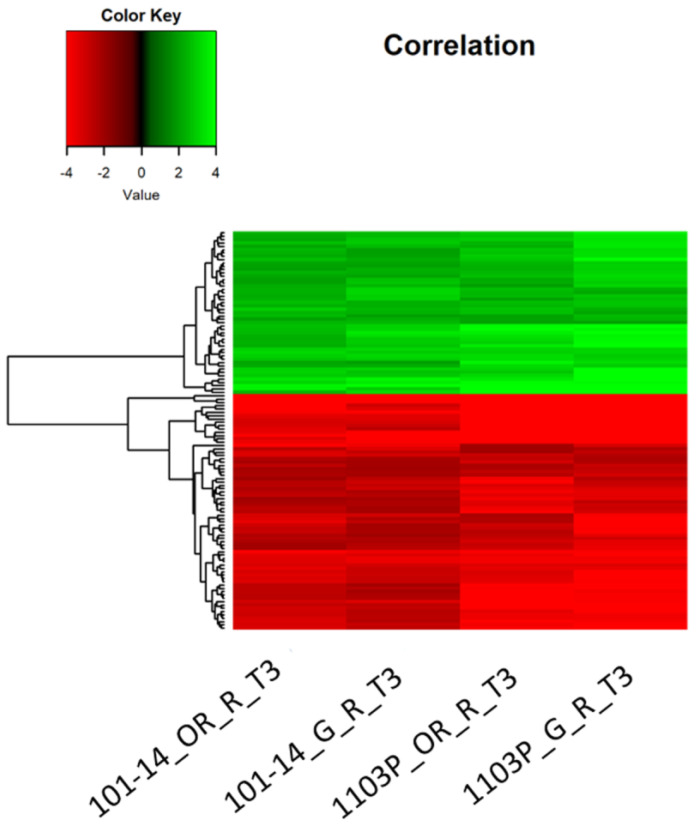
Heatmap of shared differentially expressed genes (DEGs) of 101-14MGt and 1103P grapevine rootstocks, own-rooted (OR) and grafted (G) with Cabernet SauvignoIin roots (R) at 20% soil water content (T3). Green: upregulated genes; red: downregulated genes.

Venn diagrams allowed us to identify the genes affected by drought in both 1103P and 101-14MGt. Genes with a log2 fold change value in the range higher than −2.0 and lower than 2.0 have been removed. After filtering, in most of the samples at T2, the DEGs were drastically reduced up to 0. A reasonable number of DEGs was only recorded for roots of 1103P grafted with Cabernet Sauvignon. At T3, root samples showed the highest number of shared DEGs (Figure 5). A total of 120 of common DEGs were found at T3 for both own-rooted and grafted samples of the two rootstock genotypes (Figure 5a). The heatmap of these core DEGs did not highlight divergent co-expression patterns affected either by genotypes or grafting (Figure 6). Among the up-regulated genes, there are: (i) a receptor of abscisic acid (PYL4); (ii) several germin-like proteins; (iii) two expasins (alpha and beta); (iv) some peroxidases; and (v) a thromboxane-a synthase-like protein. Among the down-regulated genes, there are: (i) sugar transporters; (ii) galactinol synthases; and (iii) tonoplast dicarboxylate transporters.

At the leaf level, the number of DEG was low, except for the grafted combination onto 1103P. Grafted and own-rooted vines showed a different response to the reduced water availability (Figure 5b). Leaves of Cabernet Sauvignon grafted onto 101-14MGt only shared 4 DEGs with leaves of the own-rooted rootstock. Similarly, only 21 DEGS were shared between the grafted and own-rooted leaves of 1103P. In general, each combination of genotype and grafting condition showed specific DEGs in both roots and leaves (Figure 5). The highest number of specific DEGs was observed in roots and leaves of the grafting combination Cabernet Sauvignon/1103P. Up- and down-regulated pathways are listed in Table 5.

### 2.5. Gene Expression Pattern of Genes Involved in ABA Metabolism in Both Own-Rooted and Grafted Plants

The expression pattern of three genes involved in the biosynthesis, signaling, and mobilization of ABA (*VviNCED2*, 9-cis-epoxycarotenoid dioxygenase 2; *VviABF2*, abscisic acid responsive element-binding factor 2; *VviBGLU12*, *V. vinifera* beta-glucosidase 12) were investigated in leaf and roots samples of 1103P and 101-14MGt rootstocks, own-rooted and grafted with Cabernet Sauvignon, and collected at 80 and 20% SWC.

**Figure 7:**
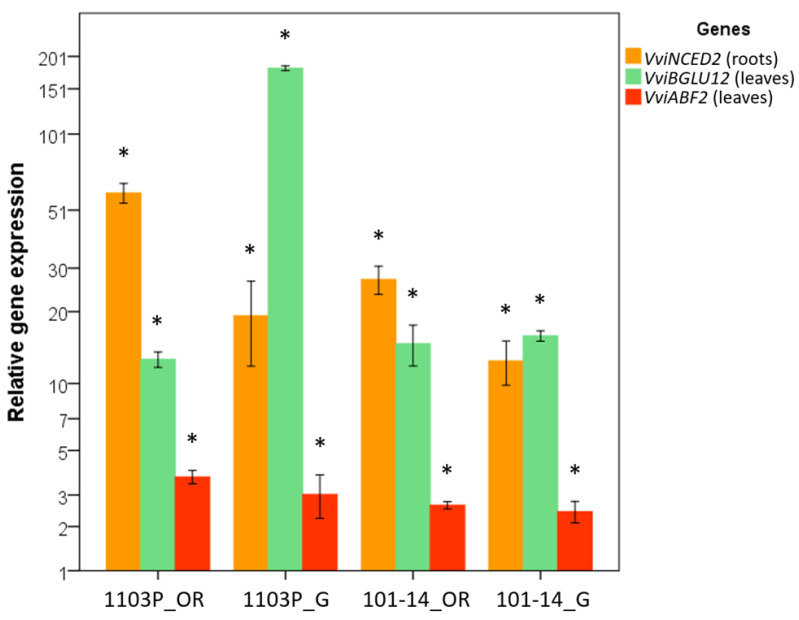
Gene expression pattern of genes involved in ABA metabolism in own-rooted (OR) and grafted (G) plants of 1103P and 101-14MGt rootstocks under drought (20% SWC) in comparison to well-watered conditions (collected at each timepoint). *VviNCED2*: 9-cis-epoxycarotenoid dioxygenase 2; *VviABF2*: Abscisic acid responsive element-binding factor 2; *VviBGLU12*: beta-glucosidase 12. *VviNCED2* was evaluated on roots, and *VviABF2* and *VviGLU12* were evaluated on leaves. The relative gene expressions of samples at control conditions (80% SWC) reaching values around 1 are omitted. Bars represent the standard deviation. Bars followed by asterisks indicate significant differences from the values recorded at 80% SWC (* *p* value = 0.05).

In the roots, the expression of the *VviNCED2* gene increased under drought stress conditions (20% SWC) in comparison to the WW (well-watered) condition in each genotype and grafting condition (Figure 7). Furthermore, *VviNCED2* showed a higher expression level in the own-rooted combinations than the grafted ones, with 1103P roots showing the highest value. In leaves, the expression of *VviBGLU12* and *VviABF2* genes increased under drought stress condition (20% SWC) in comparison to the WW condition (80% SWC) in each genotype and grafting condition (Figure 7). *VviBGLU12* was the gene that showed the highest expression level under drought conditions, with leaves of Cabernet Sauvignon grafted onto 1103P reaching the highest value. Slight differences were observed among leaf samples for *VviABF2* gene expression.

## 3. Discussion

### 3.1. Two Rootstock Genotype Models for Water Deficit Response

In this study, two rootstocks with different tolerance levels were exposed to short-term water deficit. Own-rooted 1103P and 101-14MGt rootstocks reported a similar performance under WW conditions (80% SWC), although higher gs, E, and ABA in roots were recorded by 1103P (Figure 1b and Figure 2c–h). This difference could be ascribed to the different genetic background of the two rootstocks [32]. At 50% SWC, the water deficit level seemed not to be limiting for both genotypes in terms of gas exchange and Ψs, suggesting the absence of water stress in these conditions (Figure 1). Nevertheless, the relative importance of rootstock genotype increased for several traits, such as Ψs, E, and WUE, as shown by the analysis of variance (Table 3). Thus, rootstock genotype became the main factor in determining the physiological activity of vines under severe water deficit (20% SWC), representing the major source of variability for several phenotypic traits. It is worth noting that the physiological activity of rootstocks under water deficit could be influenced by the pot size, which affects root development [33].

In response to progressive water deficit, different adaptation strategies were observed by the two genotypes. Own-rooted 1103P was able to sense water deficit and to induce stomatal closure to its leaves, reducing the loss of water by transpiration. Under severe water deficit, own-rooted 1103P reduced WUE and iWUE, due to a reduction of Pn in relation to E and gs, respectively (Figure 2b–f). Stomatal closure could be induced by the drop of Ψs, as suggested by the significant regressions observed between water potential and gas exchange. As a response to water potential drop, own-rooted 1103P also increased the level of ABA in leaves (Table 1), which probably contributed to stomatal closure. The strategy adopted by 1103P in this study under water deficit was usually referred in the literature as “avoidance” [34]. The avoidance strategy could be suitable for long term drought periods, because it reduces the risk of embolisms and allows the physiological activity to restart when environmental conditions become more favorable [35]. Moreover, this strategy reduces water use and saves water for longer periods. A similar response of 1103P to drought was reported in the literature in comparison to M4 rootstock: under mild to severe water deficit 1103P reduced gs, resulting in lower E, Pn, WUE, and water potential than M4 [36]. Due to its ability to sense and avoid water stress, 1103P was generally considered to be tolerant to drought [27,28]. Nevertheless, the avoidance strategy can limit both vegetative growth and grape ripening during the drought period, due to the inhibition of carbon assimilation.

A different behavior was shown by own-rooted 101-14MGt, which is commonly considered susceptible to water deficit [22,29]. Under water deficit, the 101-14MGt genotype reduced Ψs and gas exchange in response to severe water deficit, though higher levels were maintained compared to own-rooted 1103P. Gas exchange and ABA concentration in both leaves and roots were not significantly affected by the reduction of water potential, as reported in Table 1, suggesting that own-rooted 101-14MGt adopted a “tolerance” strategy. This strategy can be suitable for short-term drought periods since photosynthesis can support the vegetative growth and the grape ripening. Nevertheless, if drought persists for long periods, the maintenance of gas exchange can lead to vessel embolisms, deeply affecting the hydraulic conductance and presents the risk of losing water and to lead to more extreme water stress [35].

### 3.2. Grafting Combinations with V. vinifera Reflects the Rootstock Responses

When Cabernet Sauvignon was grafted onto the two rootstocks, no significant differences in gas exchange and Ψs were observed under WW conditions (Figure 2). Additionally, [32] also found no effect of the rootstock genotype on Cabernet Sauvignon water status, comparing Ramsey (*V. champinii*) and Riparia Gloire de Montpellier (*V. riparia*) as a rootstock.

Differences appeared at 50% SWC. The two grafting combinations responded differently according to the first component of PCA (Figure 3b). Under severe water deficit, the trend of Ψs, gs, E, Pn, WUE, and iWUE observed for own-rooted 1103P and 101-14MGt was confirmed in grafted plants (Figure 1 and Figure 2). In fact, the reduction of Ψs was significantly related to the drop of gas exchange only when Cabernet Sauvignon was grafted onto 1103P (Table 2). A similar response was found in the literature for Cabernet Sauvignon grafted onto rootstock SO4, which reduced gs in response to Ψs [37]. Results suggested that rootstock 1103P induced in Cabernet Sauvignon an “avoidance” behavior in response to water deficit, whereas the grafting combination with 101-14MGt adopted a “tolerance” strategy. Nevertheless, differences between the two rootstocks in terms of Ψs and gas exchange seemed to be less pronounced when they were in grafting combination with Cabernet Sauvignon, probably due to the contribution of the scion. The two grafting combinations reported differences in terms of ABA dislocation. In fact, under severe water deficit (20% SWC) 101-14MGt increased the level of ABA in the leaves, maintaining the same level of ABA in the roots (Figure 2g). On the other hand, under water deficit, 1103P increased the level of ABA in the roots (Figure 2h). In response to a potential water drop, the level of ABA significantly increased in Cabernet Sauvignon leaves when grafted onto 101-14MGt (Table 2). A similar trend was not observed when the scion was grafted onto 1103P, due to the high level of ABA observed in leaves under non-limiting water availability (80% and 50% SWC). High levels of ABA in leaves under WW conditions (Figure 2g) may be a consequence of the “avoidance” behavior reported in the literature for 1103P [36] and sometimes for Cabernet Sauvignon [38]. The hypothesis is that this combination of scion and rootstock induced accumulation in leaves of the inactive ABA-GE before the stress occurred, in order to rapidly reduce gs. Thus, stomatal closure for this grafting combination could be induced by the de-glycosylation of the ABA already in the leaves, rather than an increment of ABA production [39]. Generally, the two rootstocks showed different strategies in response to mild and severe water deficit, especially at hydraulic (Ψs) and hormonal (ABA in leaves and roots) levels, adopting the same strategies when grafted with Cabernet Sauvignon.

### 3.3. Roots Are the More Responsive Organ in Perceiving the Water Deficit

The highest number of DEGs was observed at severe water stress (20% SWC), in both rootstocks, in both grafting conditions and in both tissues (Table 4), with values ranging from 3.8k to 7.1k DEGs in roots and from 1.7k to 2.8k DEGs in leaves. These values seem to not be affected by the mapping procedure, although rootstock reads were mapped onto the *vinifera* transcriptome.

The root system is crucial for plant development. Under our experimental conditions, roots seemed to be the more responsive organ than leaves for both rootstocks and in both own-rooted and grafted conditions. Roots are the first organs to sense the soil water deficit and they transfer the signal to the shoot, regulating water use and vegetative growth [40]. The importance of roots in drought sensing is confirmed in our study by the largest number of GO terms being involved in “detection of stimuli” (Figure 4). If the number of DEGs with a log2 fold change value higher than 2.0 and lower than −2.0 between roots of own-rooted and grafted 101-14MGt plants (203 versus 205) is almost similar, the grafting seemed to affect the response of the 1103P roots, where the number is higher in the roots of grafted plants (644) than in the own-rooted (467) ones. These data suggest that the behaviour of 1103P rootstock, more than 101-14MGt, is influenced by scion (Cabernet Sauvignon) in perceiving and responding to drought. On the other hand, the number of DEGs at the leaf level was low in comparison to the ones detected at the root level (Figure 5b). Those data corroborate that the roots are the first organ in perceiving the stress. Moreover, leaves of Cabernet Sauvignon coming from plants grafted with 101-14MGt and 1103P shared only two DEGs, suggesting that scion is affected by rootstock. In [30,41], it was found that the scion grafted onto 1103P was found to be particularly affected by the grafting process.

### 3.4. A Core Set of Genes Is Regulated in Roots of Both Rootstock Genotypes under Severe Water Deficit

A core set of DEGs (120) has been identified to be associated with the drought response in the roots of both genotypes (101-14MGt and 1103P) and the grafted combinations (own-rooted and grafted with Cabernet Sauvignon), showing a similar behaviour in both genotypes and conditions. The up-regulated genes are involved in the ABA signaling pathway, such as PYL4 (an ABA receptor; [42]) and germin-like proteins (playing a key role in plant development and in plant defense responses against abiotic and biotic stresses; [43]), suggesting ABA involvement in response to water stress. Other differentially expressed genes are: expansins, cell wall-loosening proteins that regulate cell wall expansion and cell enlargement, and the expression of these genes can be increased by drought [44]; peroxidases, key enzymes of lignin biosynthesis, a polymer known to be involved in the drought response [45]; and a protein similar to thromboxane-a synthase, involved in the biosynthesis of strigolactones, a novel class of hormones playing a role in regulating stress tolerance to drought and salt [46]. Among the down-regulated genes, there are genes related to drought response as well. These genes are involved in the transport and biosynthesis of carbohydrates and various carboxylic acids, such as SWEET transporters, sugar transporters associated with the root elongation of plants under drought stress [47], galactinol synthases, key enzyme in the synthesis of raffinose family oligosaccharides, working as osmoprotectants [48], and tonoplast dicarboxylate transporters involved in malate accumulation [49]. Interestingly, it was demonstrated that in *Arabidopsis*, the smaller stomatal pores are a consequence of reduced accumulation of malate, acting as a signaling molecule in the control of turgor pressure within guard cells [50]. All together, these genes represent a pool of genes related to the grapevine rootstock response to drought under severe water conditions that are not affected by rootstock genotype nor grafting.

### 3.5. 1103P Shows a Greater Transcriptomic Responsiveness at the Root Level

The number of unique DEGs identified by the roots of own-rooted and grafted 1103P plants (81 and 240, respectively) was far above the ones identified in own-rooted and grafted roots of 101-14MGt (20 and 19, respectively) (Figure 5a), suggesting a higher reactiveness of 1103P in perceiving severe stress and responding to stress conditions. Out of the 81 DEGs modulated by drought in 1103P roots of own-rooted plants, AP2/ERFs (APETALA2/ETHYLENE RESPONSIVE FACTOR) and ethylene-responsive transcription factors are worth mentioning separately, due to their involvement in drought response. AP2/ERFs are involved in a wide range of stress tolerance, including drought. Many of those transcription factors respond to ABA and ethylene, to activate ABA and ethylene stress-response genes [51]. The expression of these genes, involved in ABA signalling, could be the result of an ABA increment observed in the 1103P roots under severe drought conditions (Figure 2h). In the roots of 1103P grafted plants, AP2/ERF transcripts were modulated as well. Additionally, it was observed the modulation of transcripts encoding for the cell wall construction (genes involved in the root system growth, probably responsible for 1103P ability to grow a wide and deep roots system [26]), transcripts encoding for the tonoplast intrinsic proteins (involved in the uptake and retention of water into the cells [50]), and transcripts encoding for receptor protein kinases (involved in the signal transduction mechanism [52]).

In 101-14MGt roots, the transcripts, whose role is involved in the drought response, are cytochromes P450 and carotenoid-cleavage dioxygenases in both own-rooted and grafted plants. Those genes participate in stress responses, including drought [53,54]. Transcripts specific to own-rooted 101-14MGt plants are the gene coding for the dehydration-responsive protein rd22, a gene inducible by water shortage in ABA-dependent pathway [55], and a genes coding for expansins, involved in the maintenance of root growth in drought conditions [44]. While, transcripts specific to grafted 101-14MGt plants are transcripts encoding for a pectin esterase inhibitor (a key regulator of pectin methylesterase (PME), inhibiting PME activity, and involved in drought response [56]).

### 3.6. Rootstock Influences the Scion Leaf Transcriptome under Water Deficit

At the leaf level, the behavior of Cabernet Sauvignon is affected by the rootstock. Leaves of Cabernet Sauvignon plants grafted onto 101-14MGt showed an up-regulation of a gene encoding for xyloglucan endotransglucosylase protein, recognized as wall-modifying protein, as well as two kda class ii heat shock proteins, contributing to several physiological pathways, such as drought response [57,58]. Leaves of Cabernet Sauvignon grafted onto 1103P showed an up-regulation of the gene encoding for β-fructofuranosidase, able to hydrolyse the glycosidic bond of sucrose-6-phosphate in D-glucose-6-phosphate and D-fructose. These osmolytes are involved in osmotic adjustments. At low concentrations, they may have also a role as ROS scavengers and in the regulation of gene expression and metabolic processes [59]. Other genes strongly modulated by leaves of Cabernet Sauvignon grafted onto 1103P are genes involved in the synthesis of flavonoids. The synthesis of flavonoids is one of the plant defence mechanisms to regulate ROS (reactive oxygen species) homeostasis and prevent oxidative stress, due to their antioxidant activities [60]. A similar up-regulation of phenylpropanoid metabolism has been highlighted by [61] in Pinot Noir plants grafted onto 1103P in comparison with the one grafted onto 101-14MGt when grown on sandy soils. Fasciclin-like arabinogalactan proteins [62], aspartic proteinases [63], and serine carboxypeptidase-like proteins [64] could be proteins involved in the response to water deficit and up-regulated on leaves of Cabernet Sauvignon grafted onto 1103P. This group of genes can explain the stomata closure that occurred on leaves of Cabernet Sauvignon grafted onto 1103P.

### 3.7. ABA Content Is Affected by Grafting under Water Deficit

ABA deserves a separate comment. ABA plays a crucial role in drought response, contributing to a reduction of transpiration by inducing stomata closure [65]. In our experimental conditions, grafting seemed to positively influence the synthesis of ABA_root_ under severe water deficit. Grafted plants increased the content of ABA_root_ during the more severe stage of imposed stress (T3—20% SWC), while own-rooted plants in the same condition decreased it. In the roots of own-rooted 1103P, an up-regulation of the gene encoding for abscisate β-glucosyltransferase (ABA-UGT) occurred. This gene acts in glycosylating ABA in ABA-glucosilester (ABA-GE). ABA-GE is critical for the homeostasis of ABA, because it is a transport and storage ABA form [39]. At the same time, the ABA catabolism genes, such as ABA 8′-hydroxylases, that deactivates ABA irreversibly and converts it into phaseic acid, were down regulated in the roots of 1103P plants, both own-rooted and grafted. Gene expression analysis showed that *VviNCED2*, a key enzyme for the de novo biosynthesis of ABA, is highly expressed in the roots of own-rooted 1103P. These data can explain the increase of ABA in the roots of 1103P. At the leaf level, ABA content was similar between own-rooted and grafted plants at 20% SWC. ABA_leaf_ content increased in own-rooted plants as the magnitude of the imposed stress increases and remained almost unchanged in grafted plants. At the transcriptomic level, several studies on ABA signal transduction allowed us to define a model of action that utilizes RCARs (regulatory components of ABA receptors), comprising PYR1/PYLs (pyrabactin resistance 1/pyrabactin resistance-like) receptors, PP2C (type 2C protein phosphatases), and SnRK2 (sucrose non-fermenting-1 (SNF1)-related protein kinase 2). PYR/PYL receptors negatively regulate PP2C, which in turn negatively regulates SnRK2 [42]. Up-regulation of PYR/PYLs and down-regulation of PP2C were observed in all samples, except for the leaves of Cabernet Sauvignon grafted onto 101-14MGt, suggesting that endogenous ABA increases the expression levels of ABA receptors. Down-regulation of genes related to ABA catabolism and over-expression of PYR/PYL receptors in 1103P roots, along with over-expression of PYR/PYL and down-regulation of PP2C in leaves, can explain the earlier and stomatal closure of 1103P compared to 101-14MGt. ABF2, a basic leucine zipper protein that regulates ABA-dependent stress-responsive gene expression, is activated by SnRK2 through phosphorylating. The expression of the *VviABF2* gene under drought conditions seemed to not be affected by genotype nor grafted condition. An alternative pathway to the de novo biosynthesis of ABA to increase ABA levels in plants is the hydrolysis of glucose-conjugated ABA by β-glucosidase. *VviBGLU12* is highly expressed in all leaf samples under drought conditions, suggesting that this pathway is activated by drought in grapevine leaves. As mentioned before, a similar pattern of up-regulation of 1103P genes related to the ABA biosynthetic pathway was found also in a previous study [30].

## 4. Material and Methods

### 4.1. Plant Material and Growth Conditions

The experiment was performed at the Department of Agricultural and Environmental Sciences (Milan) in a greenhouse equipped with supplementary light, with a 16/8 h light/dark photoperiod [daily PPFD of ∼600 µmol of photons/(m^2^ × s)] and a cooling system, with temperatures ranging from 23 °C to 28 °C [29], relative humidity ranging from 65% to 80%, and the air VPD ranging from 0.71 to 0.96 kPa. The experiment was carried out on two-year-old rootstocks, one considered resistant to drought (1103P, *Vitis Berlandieri* × *Vitis rupestris* hybrid) and the other considered susceptible to drought (101-14MGt, *Vitis riparia* × *V. rupestris*), own-rooted and grafted with Cabernet Sauvignon (*V. vinifera*). Plants, both grafted and own-rooted, were provided by Vivai Cooperativi Rauscedo (Rauscedo, Pordenone, Italy). Grafted plants were grafted using the table graft methods. Own-rooted and grafted plants were grown in 4-L plastic pots filled with a sand–peat mixture (7:3 in volume) with an added layer of expanded clay aggregate on the bottom of the pot [29]. The plants were trained on 1 m stake and placed in a randomized complete block design (three blocks). A total of 72 plants were monitored, 36 replicates per each rootstock. Among them, 18 were own-rooted and 18 grafted. During budding, the samples were maintained in WW conditions and fertilized monthly with 100 mL of OligoGreen nutrient solution containing (GREEN Italia, Canale d’Alba, Italy).

### 4.2. Irrigation Management

Once the plants reached a well-developed canopy, eighth and ninth fully developed leaves, they were treated with two irrigation treatments (plants started their vegetative season in the second half of March and the test started at the end of April). For each rootstock-scion combination, nine plants were grown under WW conditions and nine under water-stressed (WS) conditions. The WW plants were maintained at 80% SWC, watering them every day, to restore the right field capacity. The water stress was applied to WS plants by decreasing water availability. Three samplings were performed at 80, 50, and 20% SWC, named T1, T2, and T3, respectively. At each time point, three WW and three WS plants were sampled for phenotyping and transcriptome analyses (Table 6). The SWC was calculated using the gravimetric method [66]:

$$SWC\;=\;\frac{\left(fresh\;weight-dry\;weight\right)}{dry\;weight}\;\times \;100$$

where fresh weight refers to the soil weight at field capacity and dry weight to the soil dried in an oven at 105 °C for 48 h. The test started on April the 28th (T1, 80% SWC), the plants reached the 50% SWC (T2) on May the 1st, and 20% SCW (T3) on May the 5th. Soil water content levels of individual pots during the experiment were reported in Supplementary material S8.

### 4.3. Plant Phenotyping for Drought Response

At each time point, physiological parameters (gas exchanges and Ψs; stem water potential) were measured on one fully expanded leaf per plant (i.e., from the fifth to the eighth node of primary shoot). Stomatal conductance (gs; mol H_2_O m^−2^ s^−1^), photosynthetic activity (Pn; μmol CO_2_ m^−2^ s^−1^), and transpiration (E; mmol H_2_O m^−2^ s^−1^) were measured with a portable photosynthesis system (CIRAS-2, PP Systems, Amesbury, MA, USA), equipped with PLC6 (U) cuvette 18 mm circular (2.5 cm^2^ head plate), under constant saturating PPFD of 1500 µmol photons m^−2^ s^–1^, CO_2_ concentration of 380 μmol mol^–1^, block temperature of 25 °C, and relative humidity between 60% and 70% allowing ~1.5 kPa of VPD (vapor pressure deficit) inside the leaf chamber [29]. The same leaves, chosen for gas exchange measurements, were wrapped in a plastic bag supplied with aluminum foil for 1 h and then collected to measure the Ψs (bar) using a Scholander-pressure chamber (Soil Moisture Equipment Corporation, Santa Barbara, CA) [67]. Both gas exchange and Ψs measurements were taken between 11:00 am and 2:00 pm solar time. Gas exchange measurements were used to estimate: (i) the instantaneous water-use efficiency (WUE), as the ratio between carbon assimilation (Pn) and E; and (ii) the intrinsic water-use efficiency (iWUE), as the ratio between Pn and gs [68,69].

### 4.4. Abscisic Acid (ABA) Detection in Roots and Leaves

After the in vivo measurements, the whole root system and fully expanded leaves (i.e., from the fifth to the eighth node of primary shoot) were collected to perform ABA detection, using an enzyme-linked immunosorbent assay (plant hormone abscisic acid, ABA ELISA Kit; CUSABIO, Houston, TX, USA), following the manufacturer’s instructions, as reported in [70]. Roots were first washed with water and then ground. The extract used for the ELISA test was obtained from 0.5 g of frozen and finely ground tissue incubated with 4.5 mL of sample extraction buffer and shaken overnight at 4 °C and in the darkness. Leaf extracts were filtered with filter paper, while root extracts were centrifuged at 4 °C, at 12,000 rpm for 30 min. ELISA test was resolved on Sunrise absorbance microplate reader (Tecan, Switzerland).

### 4.5. Total RNA Extraction

At each time point, total RNA was isolated from the whole root system and fully expanded leaves (collected as reported above), frozen in liquid nitrogen and stored at −80 °C, for a total of 120 samples. RNA extraction was performed on 100 mg of tissue, using Spectrum™ Plant Total RNA Kit (Sigma-Aldrich, Germany), according to the manufacturer’s instructions. RNA quantification was measured on a Qubit^®^ 3.0 Fluorometer (Life Technologies, Carlsbad, CA, USA), using Qubit^®^ RNA HS Assay Kit. The quality was checked both on an Agilent 2200 Tapestation (Agilent Technologies, Santa Clara, CA, USA), using the RNA ScreenTape (Agilent Technologies) for RNA integrity detection, and on a NanoDrop 8000 Spectrophotometer (Thermo Scientific, Waltham, MA, USA), to evaluate 260/230 and 260/280 ratios. A lithium-chloride (LiCl) treatment has been performed for those samples showing a 260/230 ratio lower than 1.8 [71].

### 4.6. Library Construction and Sequencing

One μg of high-quality total RNA was used to construct 120 cDNA libraries, using KAPA Stranded mRNA-Seq Kit (Roche, Switzerland), according to the manufacturer’s instructions. Each library was barcoded using SeqCap Adapter kit A and B (Roche NimbleGen, WI). Tapestation 2200 (Agilent) was used to confirm the final size (250–280 bps), using High Sensitivity D1000 ScreenTape kit (Agilent). KAPA Library Quantification kit—Illumina (Roche, Switzerland) was used to quantify the libraries on LightCycler 480 (Roche). Illumina HiSeq 2500 platform (Illumina, San Diego, CA, USA) was used for sequencing, with paired end runs of 2 × 50 bps. Base calling and quality control were checked by Illumina RTA v1.13 (Illumina) sequence analysis pipeline. The original sequencing datasets have been deposited in the European Nucleotide Archive (ENA) with the accession number PRJEB32438.

### 4.7. Sequence Annotation

FastQC (http://www.bioinformatics.bbsrc.ac.uk/projects/fastqc/, accessed on 20 February 2023) software was used to inspect the quality of raw reads, after adapter removing. Trimmomatic 0.36 software [36] was used to trim reads showing low quality score. Reads were mapped to the v1 prediction of grapevine PN40024 reference genome, developed at Centro di Ricerca Interdipartimentale per le Biotecnologie Innovative (CRIBI) of University of Padua (https://urgi.versailles.inra.fr/files/Vini/Vitis%2012X.2%20annotations/cribi_V1_on_assembly_12X_V2.gff3.zip, accessed on 20 February 2023), using Bowtie2 [72] tool, with default parameters. It was decided to map the reads onto a *V. vinifera* reference genome, although rootstocks belong to a different species of the genus *Vitis*, to compare the transcripts of Cabernet Sauvignon and the ones of two rootstocks. Alignments were converted from BAM (Binary Alignment/Map) to SAM (Sequence Alignment/Map) format using SAMtools [73] software package and then sorted and indexed. An ad hoc python pipeline was used to count the number of reads aligned to each RefSeq mRNA. For data analysis, only transcripts with more than 5 reads were retained.

### 4.8. Identification of Differentially Expressed Genes (DEGs)

Count data were analyzed using R software [74]. An overview of similarities and dissimilarities among samples was performed using DESeq2 [75] R package, using *pheatmap* (for heatmap analysis) and *plotPCA* (for hierarchical clustering) functions. DESeq2 has been used to identify differentially expressed genes (DEGs), performing a multifactor designs method. Per each transcript, log2 fold change, *p*-value, and adjusted *p*-value were evaluated and only transcripts showing a false discovery rate (FDR)-adjusted *p*-value <0.05 were analyzed.

Gene ontology (GO) enrichment was estimated using the R package topGO version 2.26.0. [76]. The analysis was performed on DEG lists with FDR-adjusted *p*-value <0.05 and gene2GO annotation file (where the terms of biological process ontology are included) was provided by CRIBI (https://urgi.versailles.inra.fr/files/Vini/Vitis%2012X.2%20annotations/crib”_V1_’n_assembly_12X_V2.gff3.zip, accessed on 20 February 2023). The top 50 significantly enriched GO terms were retained.

Jvenn web server (bioinfo.genotoul.fr/jvenn/example.html) [77] has been used to identify the overlaps among different DEG lists. The *heatmap.2* function of *gplots* R package [78] was used to visualize gene expression and hierarchical clustering of shared DEGs.

### 4.9. Real-Time RT-PCR of Genes Involved in the ABA Metabolism

Genes belonging to ABA biosynthesis, signalling and mobilization pathways were selected according to the literature [20,79] and and their expression was investigated through a semi-quantitative real-time RT-PCR. The genes are: *VviNCED2* (9-cis-epoxycarotenoid dioxygenase 2), *VviABF2* (abscisic acid responsive element-binding factor 2), and *VviBGLU12* (*V. vinifera* beta-glucosidase 12). The genes where selected based on their role in ABA metabolism. In particular, *VviNCED2* was selected for its role in ABA biosynthesis, *VviABF2* was selected as a sort of primary element of response in the ABA signaling pathway responsible for the regulation of other downstream ABA-responsive genes, and *VviBGLU12* was chosen for its role in the mobilization of ABA.

Expression of *VviNCED2* was investigated in roots samples, where the gene is reported to be active due to drought [20], while *VviABF2* and *VviBGLU12* expression was investigated in leaf samples. Expression of the genes was evaluated at T3 on control (80% SWC) and drought stress (20% SWC) conditions. Primers for the amplification of *VviNCED2* gene and *VviABF2* gene were obtained by [20]. *VviBGLU12* (F: 5′-GCTAAGTGGGGGAGTGAACA-3′, R: 5′-CGCGTAATCTCGGAAATCAT-3′) primers were designed on the available sequences using the Primer3 Plus software [80]. The sequence of *VviBGLU12* was obtained for homology with the *Arabidopsis thaliana* annotated sequence of the gene abscisic acid beta-glucosidase 1 (XM_002285548.3). The ubiquitin [81] gene was used as reference for data normalization. Total RNA (500 ng) was reverse transcribed with SuperScript^®^IV Reverse Transcriptase (Thermo Fischer) following manufacturer’s instructions. Real-time RT-PCR was carried out on QuantStudio^®^ 3 Real-Time PCR Systems (Thermo Fischer). Each reaction was carried out in a volume of 20 µL, using 10 µL of PowerUp™ SYBR™ Green Master Mix (Applied Biosystems), 500 nM of each primer, 4 µL of cDNA diluted 1:10, and water up to the final volume of the reaction. Each reaction was performed in triplicate. The expression of each gene was calculated by comparing the 2^−ΔΔCt^ value [82].

### 4.10. Statistical Analysis

Phenotypical data were analyzed using R software. Linear regression models were used between Ψs and other phenotypic traits Ψs, Gs, Pn, E, WUE, ABA_root_, and ABA_leaf_. Data of individual vines were used for regression models. Regressions were compared between genotypes or grafting combinations, by analyzing the interaction effect (Ψs ∗ G). Analysis of variance (ANOVA) on phenotypic traits (Ψs, Gs, Pn, E, WUE, iWUE, ABA_root,_ and ABA_leaf_) was performed using two-way factorial models after checking for the assumption of normality of distribution and homogeneity of variance. Single effects and interactions were considered significant at *p* ≤ 0.05. The ratio between the variance explained by each factor and the total variance was used to assess the relative contribution of factors for each phenotypic trait [83,84]. Data were standardized for multivariate principal component analysis (PCA) according to z distribution. Biplots and line plots were produced by using ggplot2 package [85]. Significance of differences between grafting conditions or genotypes for phenotypic traits at each SWC level was assessed using one-way ANOVA models at 0.01 ≤ *p* ≤ 0.05 (*), 0.001 ≤ *p* ≤ 0.01 (**), and *p* ≤ 0.001 (***). The 2^−ΔΔCt^ values of the ABA metabolism genes were subjected to Levene’s test to assess homogeneity of variance in R software. To evaluate the differences among control samples and the ones subjected to drought stress, and LSD (least significant difference) test was performed in R on gene expression values through *agricolae* R package. Results were displayed as bar plots generated by SPSS v.25 software.

## 5. Conclusions

Vine adaptation to abiotic stresses, such as drought, is strongly affected by rootstocks, which are involved in water deficit detection and signaling. Plants display a range of physiological and biochemical responses against drought stress at cellular and whole-organism levels making it a complex phenomenon. From the physiological point of view, 1103P and 101-14MGt rootstocks responded differently to progressive drought, depending on the intensity and the length of the stress. The 1103P rootstock reduced gas exchange and stem water potential more conspicuously than 101-14MGt under water deficit, regardless of the grafting condition. Based on the transcriptomic evidence, 1103P was more reactive than 101-14MGt to water stress, especially at the root level. This reactiveness was affected by the grafting condition in terms of downstream responses at the root and leaf level. The data reported in this work suggest that these two rootstock genotypes (101-14MGt and 1103P) can represent two possible genotype models for the study of adaptation to water deficit at physiological and genetic levels.

## Figures and Tables

**Table 1:** Linear regression models between stem water potential (Ψs) and other phenotypic traits for own-rooted 101-14MGt and 1103P. a = intercept; b = slope; R^2^ = coefficient of determination. Significant regressions were reported for 0.01 ≤ *p* ≤ 0.05 (*), 0.001 ≤ *p* ≤ 0.01 (**), and *p* ≤ 0.001 (***).

Trait	Genotype	a	b	R^2^	*p* Value	Sig.
gs ^1^	101-14MGt	323.80	375.50	0.235	0.042	*
	1103P	375.30	404.40	0.575	0.000	***
Pn ^2^	101-14MGt	3.90	1.88	0.031	0.484	
	1103P	5.78	5.65	0.491	0.001	**
E ^3^	101-14MGt	8.79	4.62	0.024	0.538	
	1103P	11.93	13.44	0.656	0.047	***
WUE ^4^	101-14MGt	2.18	−0.13	0.001	0.925	
	1103P	2.79	2.24	0.293	0.020	*
ABA ^5^ leaf	101-14MGt	5.76	−3.06	0.004	0.805	
	1103P	1.92	−5.68	0.413	0.004	**
ABA root	101-14MGt	2.25	0.30	0.001	0.915	
	1103P	1.48	0.42	0.050	0.374	

^1^ Stomatal conductance; ^2^ photosynthetic activity; ^3^ transpiration; ^4^ water use efficiency; ^5^ abscisic acid.

**Table 2:** Linear regression models between stem water potential (Ψs) and other phenotypic traits for Cabernet Sauvignon grafted onto 101-14MGt and 1103P. a = intercept; b = slope; R^2^ = coefficient of determination. Coefficients in bold represent significant differences between the two grafting combinations at *p* ≤ 0.05. Significant regressions were reported for 0.01 ≤ *p* ≤ 0.05 (*).

Trait	Genotype	a	b	R^2^	*p* Value	Sig.
gs ^1^	101-14MGt	198.39	80.38	0.034	0.465	
	1103P	243.50	211.60	0.223	0.048	*
Pn ^2^	101-14MGt	2.89	−0.14	0.000	0.939	
	1103P	4.30	3.44	0.221	0.049	*
E ^3^	101-14MGt	7.67	3.55	0.077	0.263	
	1103P	9.83	9.46	0.346	0.010	*
WUE ^4^	101-14MGt	2.61	1.09	0.088	0.233	
	1103P	3.09	2.46	0.311	0.016	*
ABA ^5^ leaf	101-14MGt	**1.20**	−9.22	0.308	0.017	*
	1103P	**18.98**	14.17	0.072	0.282	
ABA root	101-14MGt	1.29	**0.25**	0.029	0.501	
	1103P	0.39	**−2.88**	0.323	0.014	*

^1^ Stomatal conductance; ^2^ photosynthetic activity; ^3^ transpiration; ^4^ water use efficiency; ^5^ abscisic acid.

**Table 3:** Relative contribution of the grafting status (grafted or own-rooted vines), rootstock genotype (101-14MGt or 1103P) and their interaction to the variability of phenotypic traits under decreasing levels of water availability (80, 50, and 20% SWC). Significant effects were reported for 0.01 ≤ *p* ≤ 0.05 (*), 0.001 ≤ *p* ≤ 0.01 (**), and *p* ≤ 0.001 (***).

Trait	SWC ^8^	Grafting (G)	RIstock (R)	Interaction (R ∗ G)	Error
Ψs ^1^	80	69.2 *	14.6	4.6	11.6
	50	13.1 *	71.4 ***	13.1 *	2.4
	20	2.7	87.6 ***	7.6	2.1
gs ^2^	80	62.6 ***	4.0	29.2 **	4.1
	50	66.3 **	23.2	5.0	5.5
	20	22.2 *	72.3 ***	1.5	4.0
Pn ^3^	80	28.5	21.5	17.8	32.2
	50	86.4 **	4.5	1.7	7.4
	20	7.5	85.9 ***	3.8	2.9
E ^4^	80	51.9 **	6.7	35.5 *	5.9
	50	30.7	50.1*	9.5	9.8
	20	20.6 *	70.2 ***	5.1	4.2
WUE ^5^	80	34.1	41.4	4.3	20.2
	50	33.3	52.2	0.1	14.4
	20	5.1	87.9**	0.1	7.0
iWUE ^6^	80	38.8	25.1	22.9	13.2
	50	1.4	73.0	4.6	21.0
	20	4.8	87.2 ***	2.0	6.1
ABA ^7^ leaf	80	35.9 *	10.3	48.1 **	5.6
	50	17.5 *	6.4	73.9 **	2.2
	20	3.8	69.6	11.1	15.5
ABA root	80	27.2 **	15.3*	55.3 ***	2.2
	50	23.9	9.3	0.1	66.7
	20	40.9 ***	30.1 ***	28.1 ***	0.8

^1^ Stem water potential; ^2^ stomatal conductance; ^3^ photosynthetic activity; ^4^ transpiration; ^5^ water use efficiency; ^6^ intrinsic WUE; ^7^ abscisic acid; ^8^ soil water content.

**Table 4:** Overview of differentially expressed genes (percentage in the brackets) between well-watered and water stressed conditions detected in roots and leaves of two grapevine rootstocks (101-14MGt and 1103P), own-rooted and grafted with Cabernet Sauvignon at three different water stress conditions. T1 = 80% SWC (soil water content); T2 = 50% SWC; and T3 = 20% SWC. “-“ = No statistically significant differences were observed among well-watered and drought water stressed samples at 80% SWC. Well-watered plants, collected at each timepoint, were considered as control conditions.

Genotype		Differentially Expressed Genes					
		T1 (80% SWC)		T2 (50% SWC)		T3 (20% SWC)	
		Roots	Leaves	Roots	Leaves	Roots	Leaves
*Own-rooted*							
101-14MGt	Up	-	-	46 (0.11)	282 (0.84)	3157 (8.21)	976 (2.50)
	Down	-	-	44 (0.10)	155 (0.38)	1900 (5.63)	831 (2.23)
	Total	-	-	90 (0.21)	437 (1.22)	5057 (13.84)	1807 (4.73)
1103P	Up	-	-	114 (0.24)	1 (0.00)	3164 (8.32)	1463 (3.91)
	Down	-	-	98 (0.22)	-	2166 (6.25)	1341 (3.56)
	Total	-	-	212 (0.46)	1 (0.00)	5330 (14.57)	2804 (7.37)
*Grafted*							
101-14MGt	Up	-	-	42 (0.08)	56 (0.13)	2509 (6.32)	1195 (3.02)
	Down	-	-	72 (0.16)	84 (0.21)	1478 (4.27)	492 (1.35)
	Total	-	-	114 (0.25)	140 (0.34)	3987 (10.59)	1687 (4.37)
1103P	Up	-	-	350 (0.82)	-	4162 (11.12)	1440 (3.93)
	Down	-	-	638 (1.50)	11 (0.02)	2978 (8.44)	846 (2.47)
	Total	-	-	988 (2.32)	11 (0.02)	7140 (19.56)	2286 (6.40)

**Table 5:** Specific differentially expressed genes and pathways in roots and leaves of 101-14MGt and 1103P, own-rooted and grafted with Cabernet Sauvignon, under water deprivation (20% soil water content).

Rootstock	Own-Rooted		Grafted	
	Up	Down	Up	Down
	*Roots*			
101-14MGt	– root growth and development – volatile compounds biosynthesis – response to dehydration – ABA-regulated responses	– lignin biosynthesis – brassinosteroid biosynthesis – oxidative stress metabolism	– ROS production – transcription factors – biosynthesis of alkaloids	– pectin esterase inhibitor – extension protein
1103P	– polyphenol biosynthesis – terpene biosynthesis – lignin degradation – zinc transporters	– ethylene-responsive transcription factors – MYB transcription factors	– cell wall construction – tonoplast intrinsic proteins – signal transduction	– transport and storage proteins
	***Leaves***			
101-14MGt	– biosynthesis of volatile compounds – protein kinases – shikimate pathway	– heat shock protein – expansin-like protein	– heat shock proteins – cell wall construction	– stress-induced hydrophobic peptide
1103P	– receptors or proteins regulated by hormones – lignin degradation – alkaloid biosynthetic – leaf growth – cell water retention – synthesis of cell-wall proteins	– heat shock proteins	– phenylpropanoid pathway – proteolytic enzymes – aquaporins – glucanases – auxin- and gibberellin-induced proteins – promotion of cell adhesion.	– redox-regulating protein

**Table 6:** Experimental design of water stress experiment carried out on 101-14MGt and 1103P grapevine rootstocks own-rooted and grafted with Cabernet Sauvignon (CS).

Rootstock	Grafting	Treatment	T1 ^1^	T2 ^1^	T3 ^1^
101-14MGt	own-rooted	well-watered	80%	80%	80%
		water stressed	80%	50%	20%
	grafted with CS	well-watered	80%	80%	80%
		water stressed	80%	50%	20%
1103P	own-rooted	well-watered	80%	80%	80%
		water stressed	80%	50%	20%
	grafted with CS	well-watered	80%	80%	80%
		water stressed	80%	50%	20%

^1^ soil water content.

## Data Availability

The original sequencing datasets have been deposited in the European Nucleotide Archive (ENA) and the accession number is PRJEB32438 (https://www.ebi.ac.uk/ena/browser/view/PRJEB32438, accessed on 20 February 2023).

## Supplementary Materials

The following supporting information can be downloaded at: https://www.mdpi.com/article/10.3390/plants12051080/s1, Supplementary material S1. Linear regression plots between stem water potential (Ψs) and other phenotypic traits for 101-14MGt (in red) and 1103P (in blue) own-rooted (a; c; e; g; I; k), and in grafting combination with Cabernet Sauvignon (b; d; f; h; j; l); Supplementary material S2. PCA (principal component analysis) plot of mRNA-Seq read count table of roots and leaves of two grapevine rootstocks (101-14MGt and 1103P), own-rooted and grafted with Cabernet Sauvignon, maintained at three different water conditions. T1 = 80% SWC (soil water content); T2 = 50% SWC; and T3 = 20% SWC. Well-watered samples were kept at 80% SWC all the time. PC1 = Principal Component 1; PC2 = Principal Component 2; Supplementary material S3. Overall representation of changes in mRNA-Seq reads of roots and leaves of two grapevine rootstocks (101-14MGt and 1103P), own-rooted and grafted with Cabernet Sauvignon, maintained at three different water conditions. T1 = 80% SWC (soil water content); T2 = 50% SWC; and T3 = 20% SWC. Well-watered samples were kept at 80% SWC all the time; Supplementary material S4. List of differentially expressed genes identified in roots and leaves of own-rooted 101-14MGt and 1103P rootstocks at different water conditions. T2 = 50% SWC (soil water content); T3 = 20% SWC; Supplementary material S5. List of differentially expressed genes identified in roots and leaves of 101-14MGt and 1103P rootstocks, grafted with Cabernet Sauvignon, maintained at different water conditions. T2 = 50% SWC (soil water content); T3 = 20% SWC; Supplementary material S6. List of top 50 GO categories related to DEGs (up- and down-regulated) of root and leaf samples per two own-rooted grapevine rootstocks (101-14MGt and 1103P) at different water conditions. T2 = 50% SWC (soil water content); T3 = 20% SWC; Supplementary material S7. List of top 50 GO categories related to DEGs (up- and down-regulated) of root and leaf samples per two grapevine rootstocks (101-14MGt and 1103P), grafted with Cabernet Sauvignon, at different water conditions. T2 = 50% SWC (soil water content); T3 = 20% SWC. Supplementary material S8. Soil water content (%) levels for individual pots during the experiment.
