# Supplementary figures and images for: Physiological and Transcriptomic Evaluation of Drought Effect on Own-Rooted and Grafted Grapevine Rootstock (1103P and 101-14MGt)
Source: Plants (Basel). 2023 Feb 28;12(5):1080. doi: 10.3390/plants12051080 (PMC10005690; doi:10.3390/plants12051080)

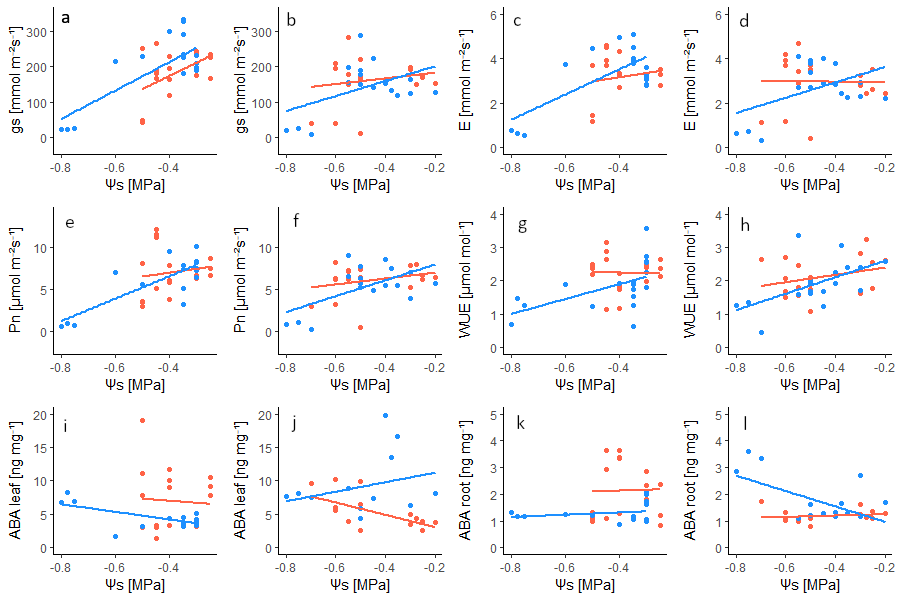

Supplement: Supplementary file 1 [file plants-12-01080-s001.zip › Supplementary material 1.tif]

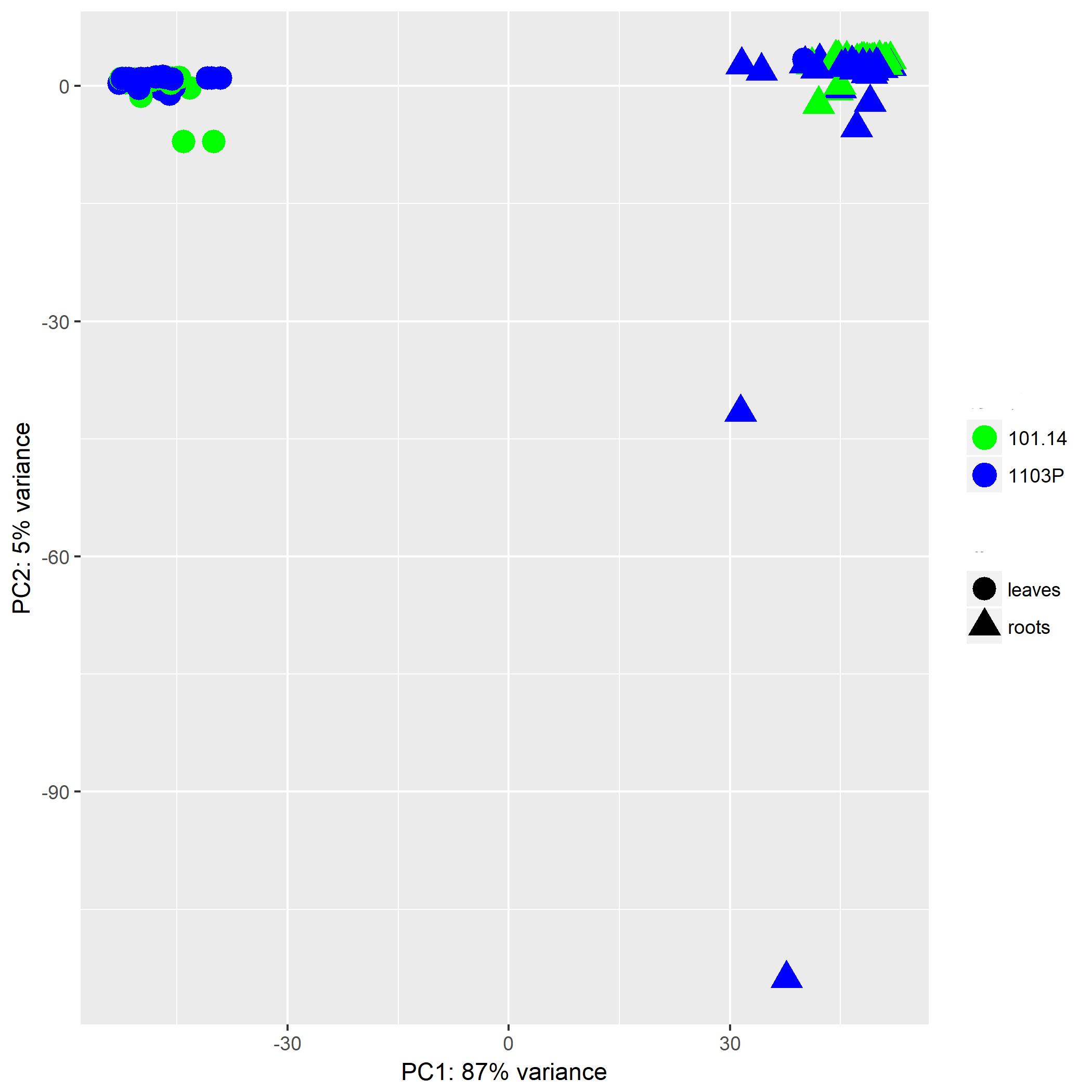

Supplement: Supplementary file 1 [file plants-12-01080-s001.zip › Supplementary material 2.tiff]

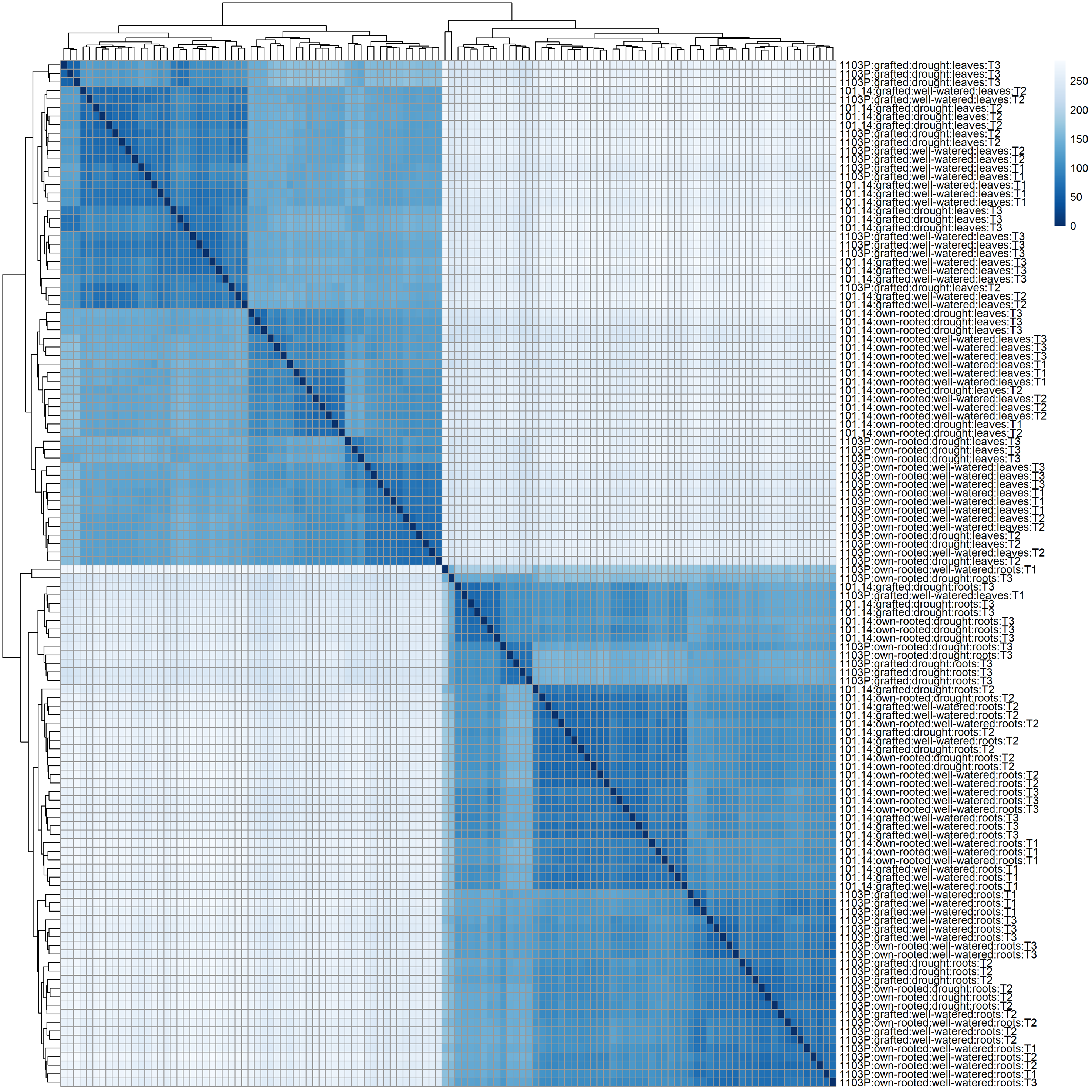

Supplement: Supplementary file 1 [file plants-12-01080-s001.zip › Supplementary material 3.tif]
